# [Corrigendum] Fisetin ameliorates atherosclerosis by regulating PCSK9 and LOX-1 in apoE^-/-^ mice

**DOI:** 10.3892/etm.2026.13343

**Published:** 2026-10-26

**Authors:** Li Yan, Qingling Jia, Hui Cao, Chuan Chen, Sanli Xing, Yan Huang, Dingzhu Shen

Exp Ther Med 21: 25, 2021; DOI: 10.3892/etm.2020.9457

Following the publication of the above article, and an Expression of concern statement (doi: 10.3892/etm.2026.13319) that was published after a concerned reader drew to the Editor's attention that one of the antibodies the authors had used in their study was apparently selected inappropriately, the authors have now responded to the Editor's request for information. The antibody (cat. no. ab51243) that the authors reported to have used in their paper is specific for the protein known as p16-ARC (or ARPC5), not the apoptosis-associated protein p16-INK4a, as was intended.

Upon re-examining their reagent records, the authors can confirm that writing “rabbit anti-p16 (cat. no. ab51243)” on line 11 of the “*Drugs and reagents*” subsection of the Materials and methods on p. 2 of this paper was a typing error. The actual antibody used in Fig. 4F for the western blot experiments was Anti-CDKN2A/p16INK4a (cat. no. ab108349). Supporting documents for the antibody purchase were provided for the inspection of the Editorial Office.

Therefore, the text in the “*Drugs and reagents*” subsection of the Materials and methods on p. 2, where the reporting of the various antibodies is described, should have read as follows (the changed text is highlighted in **bold**): “Rabbit anti-PCSK9 (cat. no. ab95478), mouse anti-p53 (cat. no. ab26), rabbit anti-p21 (cat. no. ab188224) and rabbit **anti-CDKN2A/p16INK4a (cat . no. ab108349)** were purchased from Abcam.” Note that the error made in reporting the source of the anti-CDKN2A/p16INK4a antibody did not affect the results or the overall conclusions reported in the paper. The authors express their thanks to the Editor of *Experimental and Therapeutic Medicine* for allowing them the opportunity to publish this corrigendum. All the authors agree with the publication of this corrigendum, and wish to apologize to the readership for any inconvenience caused.

